# Analysis on the Time and Frequency Domains of the Acceleration in Front Crawl Stroke

**DOI:** 10.2478/v10078-012-0028-2

**Published:** 2012-05-30

**Authors:** Joaquín Madera Gil, Luis-Millán González Moreno, Juan Benavent Mahiques, Víctor Tella Muñoz

**Affiliations:** 1 Departamento de Educación Física y Deportiva. Universidad de Valencia.

**Keywords:** swimming, Root Mean Square, power peak, power peak frequency, spectrum area

## Abstract

The swimming involves accelerations and decelerations in the swimmer’s body. Thus, the main objective of this study is to make a temporal and frequency analysis of the acceleration in front crawl swimming, regarding the gender and the performance. The sample was composed by 31 male swimmers (15 of high-level and 16 of low-level) and 20 female swimmers (11 of high-level and 9 of low-level). The acceleration was registered from the third complete cycle during eight seconds in a 25 meters maximum velocity test. A position transducer (200Hz) was used to collect the data, and it was synchronized to an aquatic camera (25Hz). The acceleration in the temporal (root mean square, minimum and maximum of the acceleration) and frequency (power peak, power peak frequency and spectral area) domains was calculated with Fourier analysis, as well as the velocity and the spectrums distribution in function to present one or more main peaks (type 1 and type 2). A one-way ANOVA was used to establish differences between gender and performance. Results show differences between genders in all the temporal domain variables (p<0.05) and only the Spectral Area (SA) in the frequency domain (p<0.05). Between gender and performance, only the Root Mean Square (RMS) showed differences in the performance of the male swimmers (p<0.05) and in the higher level swimmers, the Maximum (Max) and the Power Peak (PP) of the acceleration showed differences between both genders (p<0.05). These results confirms the importance of knowing the RMS to determine the efficiency of the swimmers regarding gender and performance level

## Introduction

The pure swimming phase involves accelerations and decelerations in the swimmer’s body, with great differences among the four competition strokes regarding their own patterns of movement (Morouço et al., 2006). Several authors (Costill, Maglischo, & Richardson, 1992; Counsilman, 1968; Counsilman, 1983; Maglischo, 1982; Maglischo, 1993; Maglischo, 2003) have described the propulsive actions of the arms, legs and body positions and their coordinations in order to reach a better performance in each of the swimming strokes. During front crawl swimming, there are three propulsive movements for each arm action (Chollet, 2004; Maglischo, 2003) with different arm to arm coordinations(Chollet, Chalies, & Chatard, 2000; Costill et al., 1992; Counsilman, 1983; Maglischo, 2003) and six, four or two kicks within each complete arm stroke (Maglischo, 2003) along with a bodyroll and a breathing pattern depending on the swimming intensity (Chollet, 2004; Maglischo, 2003). All these movements, actions and coordinations result in a number of accelerations of different magnitude for each stroke depending on their temporal coordination and propulsive efficacy.

Commonly, the intra-cycle acceleration of the swimmer’s body during swimming displacement is the result of the interaction of propulsive and braking forces, and it depends on the swimming speed (Ungerechts, 1988). Holmér (1979) found a concomitant relationship between the velocity fluctuations and the acceleration. However, the most important innovation of his study was the frequency analysis of the acceleration during swimming. Nevertheless, the most applied methodology to study the different swimming strokes regarding their acceleration has been done through the temporal analysis using either quantitative or qualitative methods. Qualitative analysis of the acceleration shows the relationship between the movements of the swimmer and the variations of the intra-cycle acceleration (Buchner & Reischle, 2003; Holmér, 1979; Mason, Tong, & Richards, 1992).

Furthermore the quantitative analyses of the acceleration have only obtained statistical parameters such as the mean, range, the variation coefficient or using the Root Mean Square (RMS) as the efficient value of the acceleration. The main objectives of these studies have been to describe the swimming strokes (Buchner & Reischle, 2003; Holmér, 1979; Mason et al., 1992; Slawson et al., 2008), to relate the acceleration with the swimming velocity (Holmér, 1979; Tella et al., 2008; Tella et al., 2010), to establish individual or performance differences (Slawson et al., 2008)and to analyze the changes produced by fatigue (Tella et al., 2008).

Since Holmér (1979) first used this type of analysis to show the frequency spectrum and to calculate the magnitude of the main peak (PP) and its associated frequency (PPF), until several years ago when the frequency variables of the acceleration have not been studied at the swimming strokes (Madera et al., 2010; Tella et al., 2008; Tella et al., 2010). It is since Tella et al. (2008) work when, besides incorporating the calculation of the spectral area (SA) as the total power of the spectrum, the different types of spectrum are associated to front crawl swimming.

Thus, the qualitative analysis of the frequency spectrum allows identifying different types of spectrums that are associated to a swimming stroke, and the quantitative analysis leads to calculate the main parameters associated with the spectrum (i.e. Power Peak or PP, Power Peak Frequency or PPF and SA).

Both the accelerometers (Holmér, 1979; Tella et al., 2010) and the position transducers (Tella et al., 2008; Madera et al., 2010) have been used to obtain the acceleration data. The accelerometers directly register the acceleration, but the position transducers differentiate two times their data to obtain the acceleration. Nonetheless in both cases the signal must be filtered to collect the frequencies of interest for the study of the acceleration. Different band-pass filters have been used to study the acceleration in front crawl swimming. Thus, Holmér (1979) used a band pass filter of 0.25–10Hz to emphasize the accelerations produced by the effect of the cyclical actions of this stroke (i.e. bodyroll and arm strokes) at frequencies near to 1HZ; Tella et al. (2008), with a band-pass of 1–20Hz, suggested that the accelerations with frequencies higher than 10Hz were negligible, and Madera et al. (2010) with a band-pass filter of 1–10Hz focused the study on the accelerations that may characterize the intra-cycle behavior of the front crawl swimming. As the RMS values increase and at the same velocity, the swimming stroke becomes more efficient (Holmér, 1979). Also the RMS shows a positive relation with the velocity, and it lessens with the apparition of fatigue (Tella et al., 2008). In a more recent work (Madera et al., 2010) it is shown that the swimmers that concentrate their accelerations into only one frequency (frequency spectrums with only one PP) swim faster and obtain higher values of RMS.

The frequency spectrums may inform about either the relative contribution of the propulsion of arms and legs (Holmér, 1979) or the sequence of propulsive forces during swimming (Tella et al., 2008). So Holmér (1979) established that the main PP is provoked by the arms action, and the second peak is associated to the movement of the legs. However, Tella et al. (2008) and Madera et al. (2010) suggest that only one peak at only one frequency may reflect the propulsive actions of both arms and legs. These authors also show different types of spectrums (i.e. one PP, two PPs or more than two PPs) that represent higher or lower entropy at the frequencies of the propulsive actions in front crawl swimming.

Thus, Holmér (1979) interprets that the PP of the frequency spectrum represents the accelerations of the movement of the arms (PPF<1Hz) and that an increase of the swimming speed provokes the emergence of another peak in the spectrum, associated to the main one (PPF≈2Hz) and that is related with the increase of the movement of the legs. This explanation is questionable due to the results obtained by Tella et al. (2008) and Madera et al. (2010) that classify the spectrums depending on the existence of one, two or several PPs. All these spectrum patterns have a PPF near 6Hz, which could represent the accelerations of arms and legs during a complete stroke.

So the temporal and frequency analysis of the acceleration may be characterizing its efficacy (RMS) and its structure (PP, PPF and SA). However, there is a lack of studies that analyze the presence of these profiles regarding the performance level of the swimmer, not the velocity. It seems reasonable the hypothesis that states that those profiles with less entropy, that is with a higher concentration of accelerations associated to one frequency (i.e. only one PP) may distinguish the better swimmers from the rest. Furthermore, neither of the studied temporal and frequency parameters have been characterized to establish possible differences between performance levels in swimmers.

Therefore, the proposal for this study is to know the behavior of the acceleration in front crawl swimming concretely by analyzing the types of spectrums, the temporal and frequency parameters of the acceleration regarding the gender and the performance.

## Materials and Methods

### Participants

The knowledge of the profiles, the performance level identification and the differences between the temporal and frequency parameters of the acceleration may be useful tools for coaches to design new objectives when planning the training program. Therefore, it has been done a descriptive analysis of the different types of spectrums and of the temporal and frequency parameters of the acceleration regarding the gender and performance level.

After having signed an informed consent, 51 regional and national front crawl swimmers from different clubs (age 17.06 ± 0.42 years; weight 63.22 ± 1.52 kg; height 172.52 ± 1.42 cm) took part in this research study. The swimmers neither suffered musculoskeletal pathologies nor restrictions, which hindered their performance during events. All the procedures described in this study fulfilled the requirements listed on the Helsinki Declaration of 1975 and its later amendment in October 2000.

### Procedures

After a standard warm up (25–30 min), swimmers performed a 25 m front crawl at maximum speed with water start.

### Measures

The studied dependent variables in the temporal domain were the mean velocity (V), stroke frequency (SF), root mean square (RMS) and the minimum (Min) and maximum (Max) values of the acceleration. In the frequency domain, the observed variables were the peak power (PP), the power peak frequency (PPF) and the spectral area (SA).

The analyzed dependent variables were the gender and the performance level (L). The performance level was set considering the best result of the 2007–8 season in the 100 meters freestyle and its points, taking into account the Spanish national record of his/her gender and age group The swimmers were grouped according whether or not the 700 points barrier was surpassed, where L1 (59.48±4.06 sec) surpassed the 700 points and L2 (64.40±6.24 sec) did not surpass them.

Acceleration was differentiated from the position–time data recorded using a position transducer (SignalFrame, SportMetrics®, Valencia, Spain), recording at 200 Hz. The apparatus consisted in a resistive sensor (i.e. which produced a resistance of 250 g) with a coiled cable that was fastened to the swimmers’ waists by means of a belt. The swimmers started the test sets from inside the swimming pool. The position transducer data was converted from analogue to digital (A/D) with a signal conditioner (Sportmetrics®).

Three complete stroke cycles after the third cycle were recorded using an underwater video camera, perpendicular to the swimmer’s plane of displacement, recording at 25Hz (from the first to the third entry of the right or left hand). Stroke frequency (SF; Hz) was calculated from this data.

**Figure 1:**
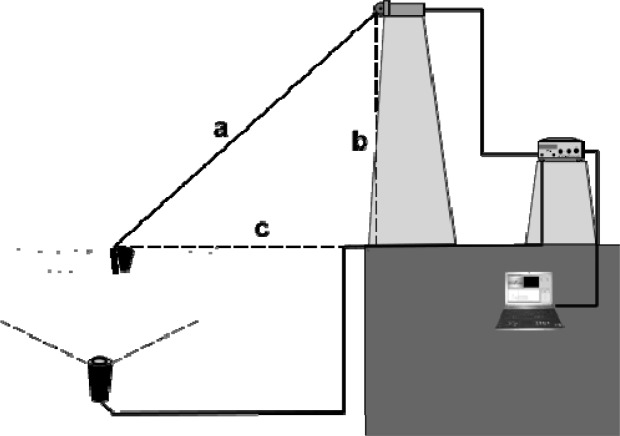
Schema of the position transducer and the swimmer during the test

The position transducer was set on the vertical edge of the pool at 2m height from the surface of the water (figure 1) with the purpose of avoiding any interference in the propulsive actions of the legs with the cable.

To obtain the swimmers’ position, the following formula was applied:

$$c=\sqrt{a^{2}-b^{2}}$$

Where *a* is the position of the cable during the displacement of the swimmer, *b* is the height of the position transducer (2m) and *c* is the position signal that represents the displacement of the swimmer (figure 1).

The position signal was differentiated two times to obtain the corresponding acceleration data (ms^−2^). The first differentiation corresponds to the following formula:

$$v=\frac{s_{2}-s_{1}}{t_{2}-t_{1}}=\frac{\Delta s}{\Delta t}$$

Where *v* is the velocity, *s* is the space and *t* is the time.

The second differentiation corresponds to the next formula:

$$a=\frac{v_{2}-v_{1}}{t_{2}-t_{1}}=\frac{\Delta v}{\Delta t}$$

Where *a* is the acceleration, *v* is the velocity, *s* is the space and *t* is the time.

To analyze the position signals, a specific program was written and run in Matlab 7.1 (R14) (Mathworks Inc., Natick, USA). The position signal was differentiated two times to obtain the corresponding acceleration signal (ms^−2^). The acceleration signal was filtered using a fourth-order Butterworth filter to create a band-pass filter of 1–10 Hz, so the analysis was focused on the intra-cycle accelerations. This signal was then analyzed in both the time and frequency domains.

Given the fact that the amplitude of the acceleration was unstable in the first and final seconds of each swimming set, the eight central seconds were selected in each trial to analyze the signal (Caty et al., 2007).

The signal amplitude was examined in the time domain with a root mean square (RMS), and processed in 100 ms sized bins. The RMS value is given by the following formula:

$$x_{\mathrm{RMS}}=\sqrt{\frac{x_{1}^{2}+x_{2}^{2}+\cdots x_{n}^{2}}{n}}$$

The frequency spectrum amplitude was analyzed with the periodogram method (Pollock, 1999), which permits to discover the hidden frequencies in a signal. This was performed by using the Matlab SPECTRUM function, and averaged with the Welch method. A 1024-point Hamming window was used for this purpose. The dependent variables calculated in the frequency domain were: the peak power (PP; the highest value of the power spectrum), the peak power frequency (PPF; the frequency associated with the peak power) and the total power contained in the spectrum area (SA), which is the total power of the whole spectrum between 0 and 10 Hz, in which the area under the power spectrum has been attenuated between 0 and 1Hz.

The images captured on video were used to obtain cinematic values for the swimming set. Eight seconds after the third stroke were selected (Caty et al., 2007). This event was used to calculate the space–time data corresponding to a swimming series. The mean velocity (V) was obtained with the position transducer and the stroke frequency (SF) was calculated using the video.

### Statistical analysis

The statistical analysis was performed with the SPSS software, version 15.0 (SPSS Inc., Chicago, IL, USA). All variables were verified for normality using a K–S normality test.

Standard statistical methods were used to obtain the descriptive statistics (mean, SEM), and a one-way ANOVA was applied to establish differences between groups. Scheffe post hoc tests were used to determinate specific differences between means. All differences with *p*≤0.05 were accepted as statistically significant and those with *p*≤0.01 as very significant.

## Results

### Different types of spectrum

By observing the obtained acceleration graphs, two patterns have been detected.

**Figure 2:**
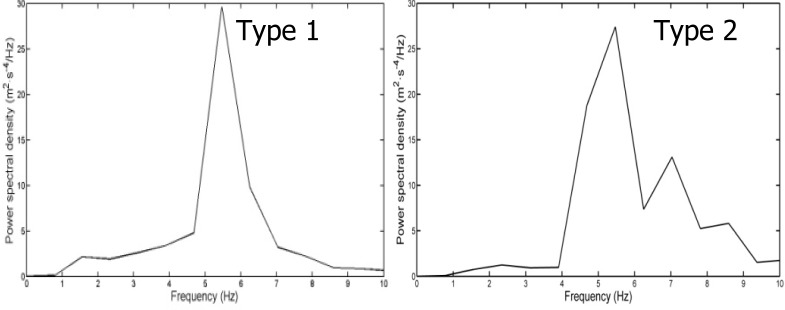
Examples of two spectrums of type 1 and 2 in front crawl swimming

Thus, the spectrums have been grouped into these two types: the ones with one main peak and low entropy, and the ones with more than one peak and higher entropy or with a wide range of frequencies in the main peaks. Figure 2 shows examples of the different types of spectrum.

**Figure 3:**
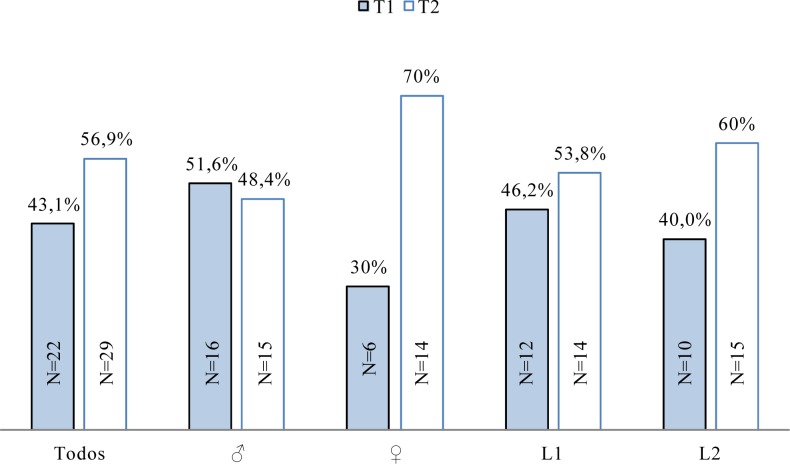
Distribution of the different types of spectrums regarding gender (♂ and ♀) and level (L1 and L2), representing the number (N) and the percentage (%) of the type 1 (T1) and type 2 (T2) spectrums

Figure 3 shows the spectrum distribution regarding gender and performance.

### Differences regarding gender, performance level and gender and performance level

#### Differences regarding gender

When comparing the different variables between genders, the results show significant differences in the temporal (F_7.41_=12.312; p<0.001) and frequency (F_3.45_=3.652; p=0.019) domains.

The temporal domain variables V (F_1.47_=92.765; p<0.001), RMS (F_1.47_=5.100; p=0.029), Min (F_1.47_=5.373; p=0.025) and Max (F_1.47_=4.567; p=0.038) showed significant differences when comparing both genders (Table 1). In the frequency domain, only the SA (F_1.47_=5.740; p=0.021) showed significant differences between genders (Table 1).

### Differences between performance levels

There were differences (F_7.41_=3.349; p=0.006) in the temporal domain. The V (F_1.47_=21.077; p<0.001) was the only variable that showed significant differences when comparing performance levels (Table 2). There were no significant differences in the frequency domain when comparing performance levels (Table 2).

### Differences regarding the performance level and gender interaction

There were significant differences (F_7.41_=3.336; p=0.007) in the temporal domain when analyzing the gender and level interaction.

The RMS (F_1.47_=5.91. P=0.019) between the male swimmers of both levels and between both genders of L1, and the Max between male and female swimmers of L1 (F_1.47_=4.40. P=0.041) showed significant differences (Table 3). From the variables in the frequency domain, only the PP F_1.47_=4.65. P=0.039) showed significant differences between both genders of L1 (Table 3).

## Discussion

To our knowledge, this work is the first study that analyzes the differences between genders and performance level on the front crawl swimming acceleration in both domains (temporal and frequency). So, all the temporal variables and the PP from the frequency variables show differences between genders. Considering the performance level, the differences are not significant (p<0.05). However, in the gender and performance analysis there are only differences between male levels in RMS and PP (p<0.05). Also it is confirmed the existence of two types of frequency spectrums in the front crawl swimming.

### About the types of frequency spectrums of the acceleration

The observation of the spectrum profiles allowed us to concentrate the swimmers into different groups, regarding the number of peaks shown, similar to that of Tella et al. (2008) and Madera et al. (2010) observed in their respective works. In this study and with the intention of differentiating those spectrums that visually presented smaller variation (type 1), those spectrums with more than one relevant PP regarding the rest of the signal and those with a wide range of frequencies in the PP were considered as type 2. So the type 1 spectrums show only one PP associated to only one frequency.

This work confirms that there are different types of frequency spectrums of the acceleration in front crawl swimming. Type 1 and 2 spectrums are common in both genders and performance levels. Regarding the percentage of type 1 spectrums in this study (43.1%) it should be noted the difference with the 27.85%, observed by Madera et al. (2010). The possible cause may be the two instead of three groups that were utilized in that work.

The analysis of the spectrum profiles have been done by several authors with the aim of explaining them. Holmér(1979) identified the front crawl spectrum with a low SF (PPF<1Hz) and suggested that it represented the propulsive actions of the arms. Furthermore, this author identified several smaller peaks (between 1.5 and 10 Hz) and suggested that these may correspond to the movement of the legs. As it has been used in previous studies, the methodology in this work has reduced the registered accelerations (Madera et al., 2010: 1 to 10Hz; Tella et al., 2008: 1 to 20Hz) to frequencies lower than 1Hz by filtering the signal with a band-pass filter of 1–10Hz, after considering that the accelerations below 1Hz would represent the global actions of a complete cycle (i.e. bodyroll). Also the accelerations with frequency higher than 10Hz have been reduced, because the spectrums that were found by Tella et al. (2008) did not show important peaks in those frequencies. Then to preserve only those frequencies of interest for the study, the accelerations whose frequencies were lower than 1Hz and were higher than 10Hz were reduced to achieve a representation of only the ones that could be related to intra-cycle accelerations.

Thus the analyzed spectrums may represent the propulsive actions in front crawl swimming (i.e. arms and legs) with two different types that exist at both genders and performance levels. This distribution does not confirm the hypothesis that establishes that the spectrums with only one PP at only one frequency (type 1) may represent higher level swimmers.

Given that the final performance of a swimmer may have been caused by the sum of technical, physical, anthropometrical and physiological factors, these would have a relative importance in each swimmer. That is why the criterion that has been conducted in this work to differentiate the performance level based only on the relative level of speed has not allowed understanding the causes. The methodological proposals to test the hypothesis in the aforementioned sentences should be directed to the establishment of categories of swimmers with different technical skills, and then it may be possible to confirm in future works that the type 1 spectrums would correspond to those swimmers with better skills.

So, the frequency spectrums with more than one PP may show higher entropy or more disorder when applying cyclical propulsive forces. Two reasons may be the cause of this high entropy: the incorrect coordination between the propulsive actions of the arms and the legs and the lack of repeatability of the cyclical actions. Both reasons should be considered by coaches to improve the coordinative aspects of the arms and legs, or the repeatability with the aim of getting better propulsive efficiency.

If this was the case, knowing the type of frequency spectrum of the acceleration of a swimmer may help the coaches to control and to plan the technical training program. And, depending on the frequency distribution, to apply exercises in order to improve the coordination of the different propulsive actions and the reduction of those less efficient during front crawl swimming, aiming to concentrate all the accelerations into only one frequency.

### About the values and the kinematic differences in the temporal domain

In this work, it has been established that the male swimmers obtain higher acceleration values than their female counterparts (RMS, Min, Max). When the analysis differentiates these variables as a function of gender and performance level, there are only differences in the RMS of the male swimmers (p<0.05). The female swimmers of L2 obtained higher values than the ones of L1. This non-significant difference (p<0.05) may be due to the criterion for establishing the performance levels. So, while the general criteria for differentiating levels has been to segment the swimmers depending on the swimming speed, the levels of this work have been designed regarding the relative speed to the national record of each gender and age group and its corresponding score.

Only the results by level are comparable to other studies (Madera et al., 2010; Tella et al., 2008) because the sample was also formed by swimmers of both genders and performance level. While the RMS values (L1: 5.70 ± 0.37; and L2: 5.26 ± 0.32) are similar to those from Madera et al. (2010); the higher values obtained by Tella et al. (2008) of 7.95 ± 0.62 require a comment. The interpretation made by Holmér (1979), which stated that at the same velocity, a more efficient or a higher level swimmer would generate a lower value of RMS, does not seem the reason for this difference. Given that the registering system was similar, the differences point to the higher sampling rate of the position-time signal (1KHz) and to the different band-pass filter used (1–20Hz) in Tella et al. (2008). The same criterion justifies the differences in Min and Max between these two studies.

The fact that the RMS is the only variable that shows differences between genders and between performance levels in males is emphasizing its importance to determine the relative performance of the swimmers regarding their age. With similar number of female swimmers in both groups (L1: n=11 and L2: n=9), the lack of differences in the RMS concerning gender and performance level in female swimmers may be due to the unequal number of spectrums of type 1 (n=6) and type 2 (n=14). This characteristic of the sample would limit its discussion by disallowing the interpretation of its causes or influences. This reflects the need of new studies to confirm the importance of both temporal and frequency analysis of the acceleration.

### About the differences in the kinematic variables in the frequency domain

The lack of differences between the frequency variables may be justified by the fact that this type of variables would respond to structural characteristics of the acceleration. Thus, the criterion that has been used to segment the sample has not responded to the proposed objectives. It is possible that by establishing segmentation criterion regarding the technical skill would help to identify or to characterize this type of variables that inform how the accelerations behave during front crawl swimming.

For this purpose, as Tella et al. (2009) and Madera et al. (2010) did, to extrapolate the values of PPF regarding the SF will allow describing the frequencies of the most important accelerations during a stroke cycle. As an example, L1 male swimmers have a PPF of 5.78 and a SF of 0.93; so its extrapolation to transform into the number of accelerations during a stroke cycle is 5.37. L2 female swimmers have a PPF of 5.78 and a SF of 0.93, resulting in an extrapolation of the acceleration for each stroke cycle of 5.24. In both cases, the values may correspond to the six accelerations during a stroke cycle (Counsilman & Wasilak, 1982) as a result of the propulsive actions and their coordination.

Also the registered PP values, lower than the ones from Tella et al. (2008), may be due to the evaluated distance. Also, the differences in SA between both studies may have been caused by the range of analyzed frequencies (10 Hz vs 20 Hz).

### Limitations

The main limitation of this work is linked to the employed technology, as the acceleration data that have been obtained with sensors that only register in one direction cannot discriminate which direction has caused them. This election was made to generalize the acceleration data that were obtained with a position transducer, and therefore to offer researchers and coaches who use this tool another methodology for their studies and training controls.

The second limitation refers to the categorization that has been done, attending to the obtained score relative to the speed in each gender and age group (i.e. performance). Thus, the performance is obviously influenced by a great number of variables. From our point of view, qualitative parameters should be considered in future works. Therefore, we suggest the use of different technical skill participants for future studies on this subject.

## Conclusions

To conclude, the temporal analysis of the acceleration confirms the importance of knowing the RMS to determine the efficiency of the swimmers regarding gender and performance level. Thus, the evaluation and control of the RMS during the training process may inform to the coaches of the efficiency of the propulsive actions in front crawl swimming. About the frequency analysis of the acceleration, to establish performance levels based on the velocity is not the appropriate method. So it may be recommended to study the spectrum profiles and the frequency variables from perspectives that consider the technical skills of the swimmers.

## Figures and Tables

**Table 1:** Differences between genders in the temporal and frequency domains

		Mean (SEM)	
		Men (N=31)	Women (N=20)
Temporal	SF (Hz)	0.91 (0.01)	0.88 (0.02)
	V (m·s^−1^)	1.64 (0.02)^***^	1.42 (0.02)^***^
	RMS (m·s^−2^)	5.9 (0.32)^*^	4.84 (0.33)^*^
	Min (m·s^−2^)	−18.93 (1.2)^*^	−14.76 (1.13)^*^
	Max (m·s^−2^)	19.63 (1.25)^*^	15.41 (1.49)^*^

Frequency	PP (m·s^−2^)^2^	20.6 (3.74)	13.48 (2.86)
	PPF (Hz)	5.7 (0.17)	5.66 (0.33)
	SA (m·s^−2^)^2^	60.26 (6.94)^*^	36.97 (5.47)^*^

SEM: standard error of the mean; SF: stroke frequency; V: mean velocity; RMS: acceleration root mean square; Min: minimum value of the acceleration; Max: maximum value of the acceleration; PP: power peak; PPF: power peak frequency; SA: spectrum area.

* p<0.05;

*** p<0.001

**Table 2:** Differences between performance levels in the temporal and frequency domains

		Mean (SEM)	
		L1 (N=23)	L2 (N=28)
Temporal	SF (Hz)	0.91 (0.01)	0.89 (0.02)
	V (m·s^−1^)	1.6 (0.03)^***^	1.51 (0.03)^***^
	RMS (m·s^−2^)	5.7 (0.37)	5.26 (0.32)
	Min (m·s^−2^)	−17.4 (1.3)	−17.18 (1.26)
	Max (m·s^−2^)	18.39 (1.43)	17.55 (1.4)

Frequency	PP (m·s^−2^)^2^	19.77 (4.21)	15.78 (2.88)
	PPF (Hz)	5.83 (0.25)	5.53 (0.2)
	SA (m·s^−2^)^2^	54.16 (7.44)	47.98 (6.62)

SEM: standard error of the mean; L1: level 1; L2: level 2; SF: stroke frequency; V: mean velocity; RMS: acceleration root mean square; Min: minimum value of the acceleration; Max: maximum value of the acceleration; PP: power peak; PPF: power peak frequency; SA: spectrum area.

*** p<0.001

**Table 3:** Differences regarding gender and level in the temporal and frequency domains

			**L1**		**L2**	

		Gender	Mean (SEM)	N	Mean (SEM)	N
Temporal	SF (Hz)	♂	0.93 (0.02)	15	0,89 (0,02)	16
		♀	0.89 (0.02)	11	0,88 (0,03)	9
	V (m·s^−1^)	♂	1.69 (0.02)	15	1,59 (0,02)	16
		♀	1.47 (0.02)	11	1,36 (0,03)	9
	RMS (m·s^−2^)	♂	6.62 (0.44)^*^	15	5,23 (0,42)^*^	16
		♀	4.46 (0.40)	11	5,31 (0,54)	9
	Min (m·s^−2^)	♂	−20.02 (1.73)	15	−17,91 (1,69)	16
		♀	−13.83 (1.41)	11	−15,88 (1,85)	9
	Max (m·s^−2^)	♂	21.83 (1.73)^*^	15	17,58 (1,70)	16
		♀	13.69 (1.60)^*^	11	17,50 (2,62)	9

Frequency	PP (m·s^−2^)^2^	♂	27.28 (6.54)^*^	15	14.34 (3.36)	16
		♀	9.52 (2.22)^*^	11	18.32 (5.52)	9
	PPF (Hz)	♂	5.78 (0.13)	15	5.62 (0.30)	16
		♀	5.89 (0.59)	11	5.38 (0.16)	9
	SA (m·s^−2^)^2^	♂	70.49 (10.35)	15	50.67 (8.96)	16
		♀	31.88 (6.04)	11	43.20 (9.67)	9

SEM: standard error of the mean; L1: level 1; L2: level 2; SF: stroke frequency; V: mean velocity; RMS: acceleration root mean square; Min: minimum value of the acceleration; Max: maximum value of the acceleration; PP: power peak; PPF: power peak frequency; SA: spectrum area; ♂: male; ♀: female;

* p<0.05
